# Supplementary material for: Multimodal determinants of phase-locked dynamics across deep-superficial hippocampal sublayers during theta oscillations
Source: Nat Commun. 2020 May 5;11:2217. doi: 10.1038/s41467-020-15840-6 (PMC7200700; doi:10.1038/s41467-020-15840-6)
Supplement: Supplementary file 1 — Supplementary Information [file 41467_2020_15840_MOESM1_ESM.docx]

**SUPPLEMENTARY INFORMATION**

Multimodal determinants of phase-locked dynamics across deep-superficial hippocampal sublayers during theta oscillations

Andrea Navas-Olive et al. Nature Communication


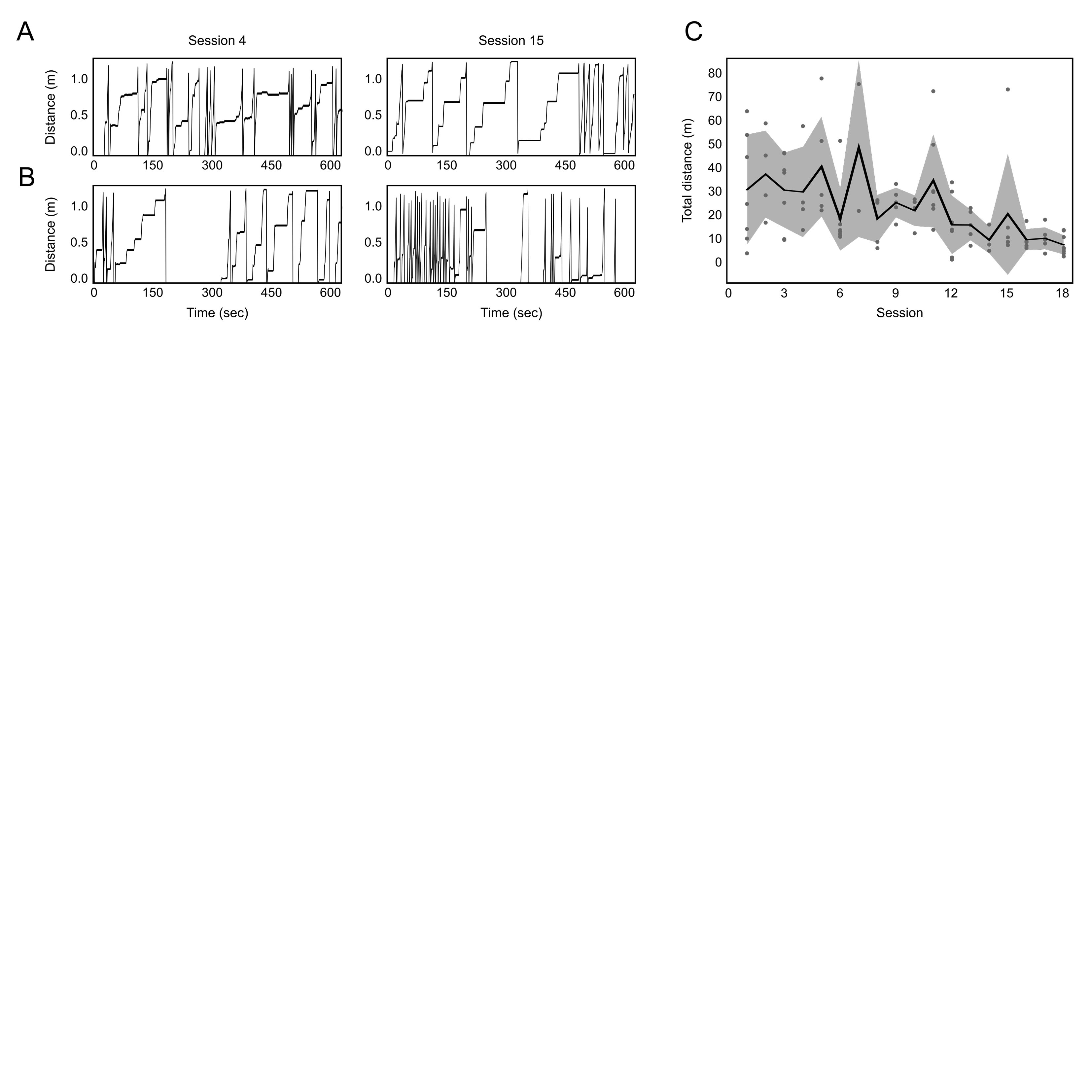


**Supplementary Figure 1. Behavior of head-fixed mice. A**, Positional data from two sessions in one mouse showing habituation to the head-fixed setup. Distance refers to position along the circumference of the wheel. Animals run a minimum of 2 sessions of 10 min each per day over 2-3 weeks to get habituated to the setup before recordings start. The animal run freely without any particular stereotyped behavior, alternating between periods of running and immobility. During immobility periods, animals are attentive to the environment, they move their forelimbs for grooming or whisk. **B**, Example of sessions 4 and 15 from another mouse. **C**, Mean ± SD total distance traveled per day in the wheel. Note animals habituate along sessions. Data from 18 sessions from 7 mice.


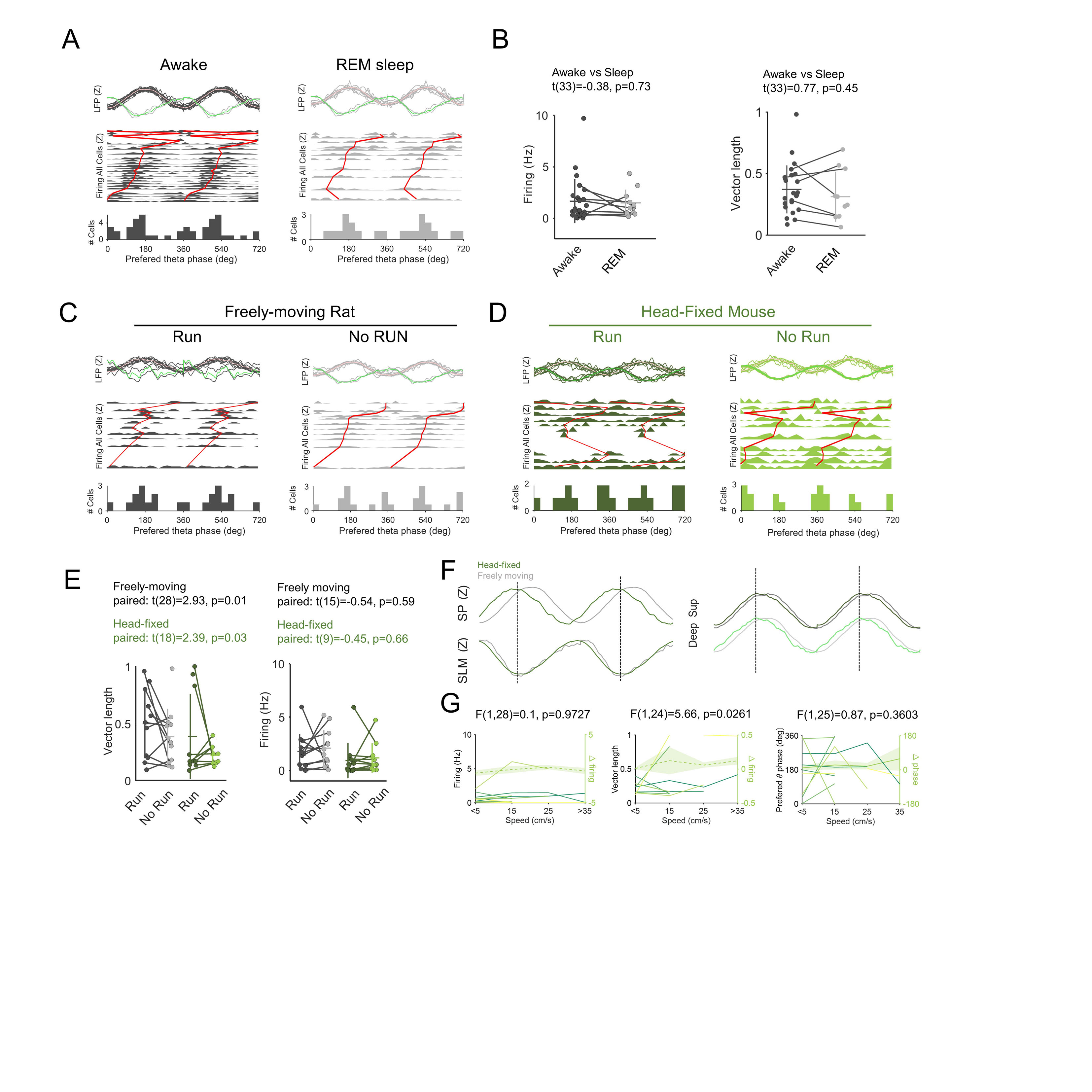


**Supplementary Figure 2. Factors influencing theta phase-locked firing. A**, Data from freely moving rats were separated in theta episodes recorded during awake (n=25 cells) and REM sleep (n=11 cells). Individual (light) and mean (bold) theta filtered LFP signals at top are z-scored (Z). **B**, No state-dependent effect in data from freely moving rats (unpaired t-test). Data from cells recorded in both conditions are linked by lines (n=8 cells). **C**, Episodes of theta oscillations from freely moving rats were separated in periods of running (> 5 cm/sec) versus periods of immobility (attentional or head-nodding theta) (n=11 cells). **D**, Episodes of theta oscillations in head-fixed mice were separated in periods of wheel running (> 5 cm/sec) versus theta periods during wheel immobility (attentional theta, whisking, forelimb movement) (n=10 cells). **E**, Statistical effects of running versus no-running periods in both preparations. Data from cells recorded in both conditions are linked by lines (n=11 cells from rats, n=9 cells from mice). **F**, Mean laminar profiles of LFP theta cycles recorded in freely moving versus head-fixed preparations, as evaluated in a subset of experiments with simultaneous SLM and SP recordings. Mean LFP signals are aligned by the SLM trough (discontinuous lines). Note phase difference (about 34º) of SP-SLM theta phase reversal between preparations (left) but not between sublayers (right). The preparation effect may be due to the use of a contralateral SLM electrode in freely moving versus head-fixed preparation. Importantly, phase-locking dynamics is reported against the local SP theta, which is similar for cells recorded from deep and superficial sublayers (right). **G**, The effect of speed was examined in a subset of cells juxtacellularly recorded from the head-fixed preparation (n=9 cells). We found some statistical effects of speed in the mean vector length but not for firing rate nor preferred theta phase of single cells.


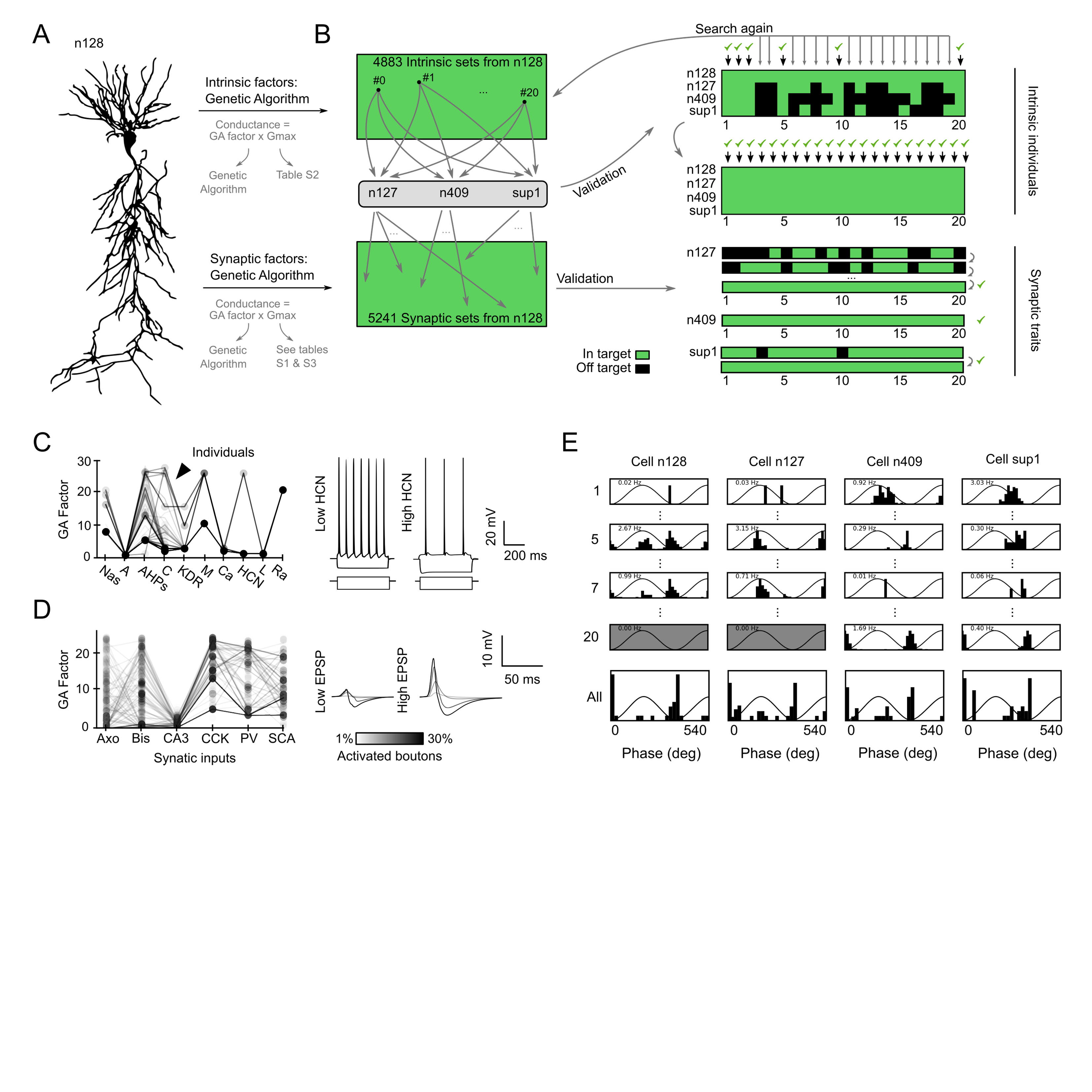


**Supplementary Figure 3. Genetic algorithms and parameter constraints.** **A**, Schematic summary of the computational strategy. For a given morphology (cell n128 from the Turner Archive in Neuromorpho) we used genetic algorithms (GA) to constraint passive and intrinsic (ionic channel maximal conductances, Gmax) as well as synaptic properties (synaptic Gmax) in a biologically realistic model of CA1 cells. **B,** Fitting by GA provides a set of values for passive, active and synaptic conductances (dubbed GA factors) resulting in realistic cellular behavior (see Fig.2B and C). We run 100 generation runs to look for parameter values that converge to the target behavior. We then chose 20 individuals (set of values) fitted in n128 and propagated them to morphologies n127 and n409 from the Turner archive and morphology sup1 (I040913C2) from the Prida archive. We next validated those intrinsic individuals that successfully fitted the experimental target across all morphologies and replaced non-valid ones by new random sets from the pool until the full dataset was validated. We proceed similarly for synaptic traits in each morphology. **C,** Solid lines link different combinations of values (individuals) giving experimentally valid input-output responses for n128, as shown in Fig.2B. Arrowhead point to one individual. Examples of current pulse responses by two different individuals are shown at right. Note validation of non-fitted intrinsic properties of CA1 pyramidal cells, such as spike afterdepolarization and sags. **D**, Same as in B for fitting synaptic responses to Schaffer collaterals stimulation with two individual examples in n128 shown at right. **E**, Examples of theta phase locked firing of different synthetic cells (individuals expressed in different morphologies) in response to a collection of glutamatergic (CA3, CA2, ECIII, ECII) and GABAergic (Axo, Bis, CCK, Ivy, NGF, OLM, PV, SCA) theta-modulated inputs (Supplementary Table 3). Mean firing rate for each synthetic cell is shown at the top of the plot. Gray shadowed cells did not meet criterion for inclusion on the analysis in Fig.2F,G (mean firing rate 0.01 - 8 Hz). The histogram at bottom shows the distribution of the preferred firing phase from all 20 cells in each morphology. Note preference for firing at the theta trough in n128 and sup1, and bimodality in morphologies n127 and n409.


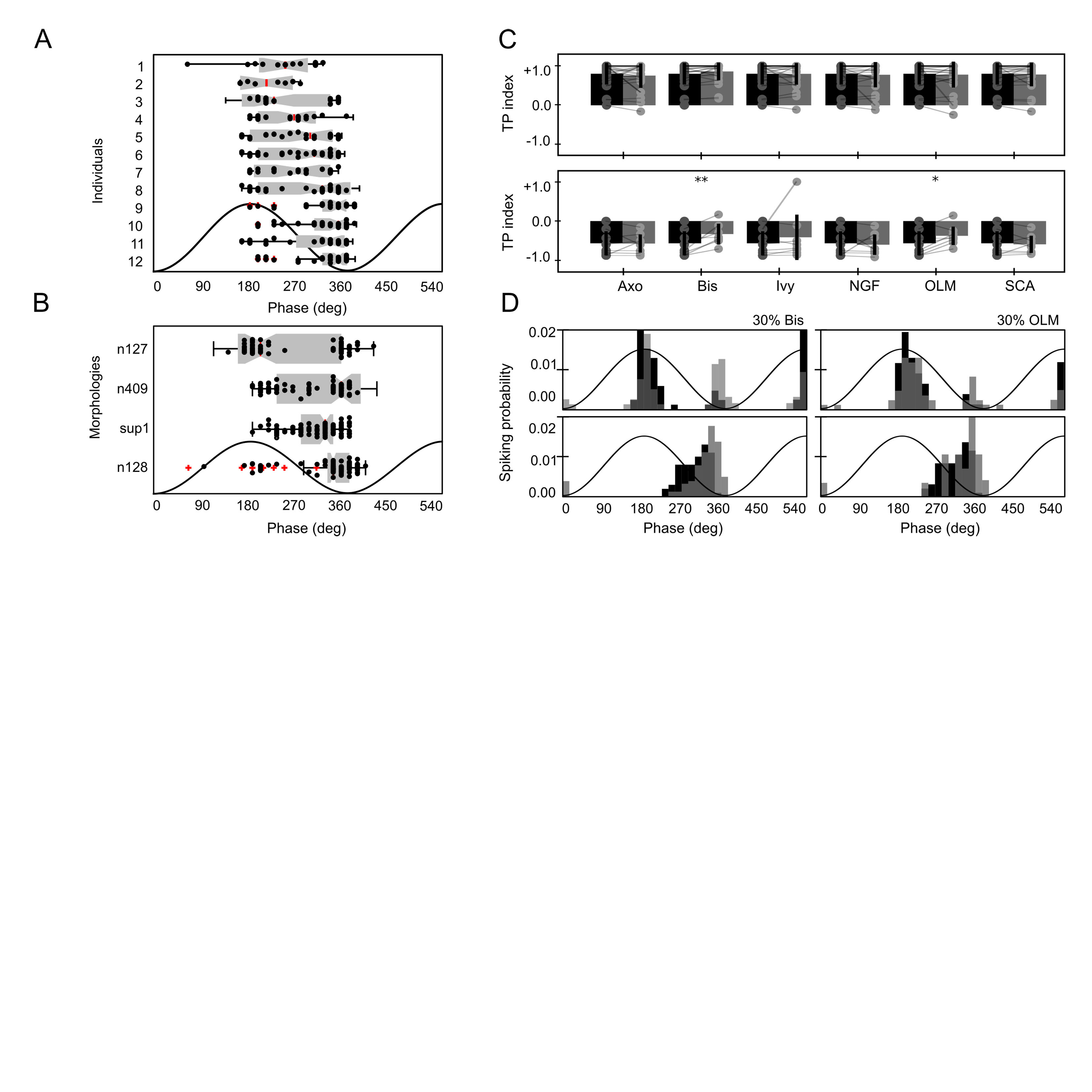


**Supplementary Figure 4. Effect of GABAergic connectivity on theta phase preference. A**, Theta phase distribution across individuals with different PV/CCK connectivity (n=48 synthetic cells; 12 individual from 4 morphologies). Individuals are ranked according to the order in Fig.2F. **B**, Effect of morphology in the distribution of preferred theta phases caused by different PV/CCK connectivity. Cell morphologies are ranked according to the order in Fig.2G. Same dataset as in A. **C**, Effect of 70% reduction of GABAergic inputs from different interneuronal types. Statistical differences were confirmed only for bistratified and OLM GABAergic inputs. Paired t-test, Bis, **, p=0.005; OLM, *, p=0.04 (n=48 synthetic cells). **D**, Representative examples of the effect of 30% reduction of GABAergic inputs from bistratified and OLM cells in the firing of synthetic pyramidal cells tuned to the theta peak (upper row) and the trough (lower row).


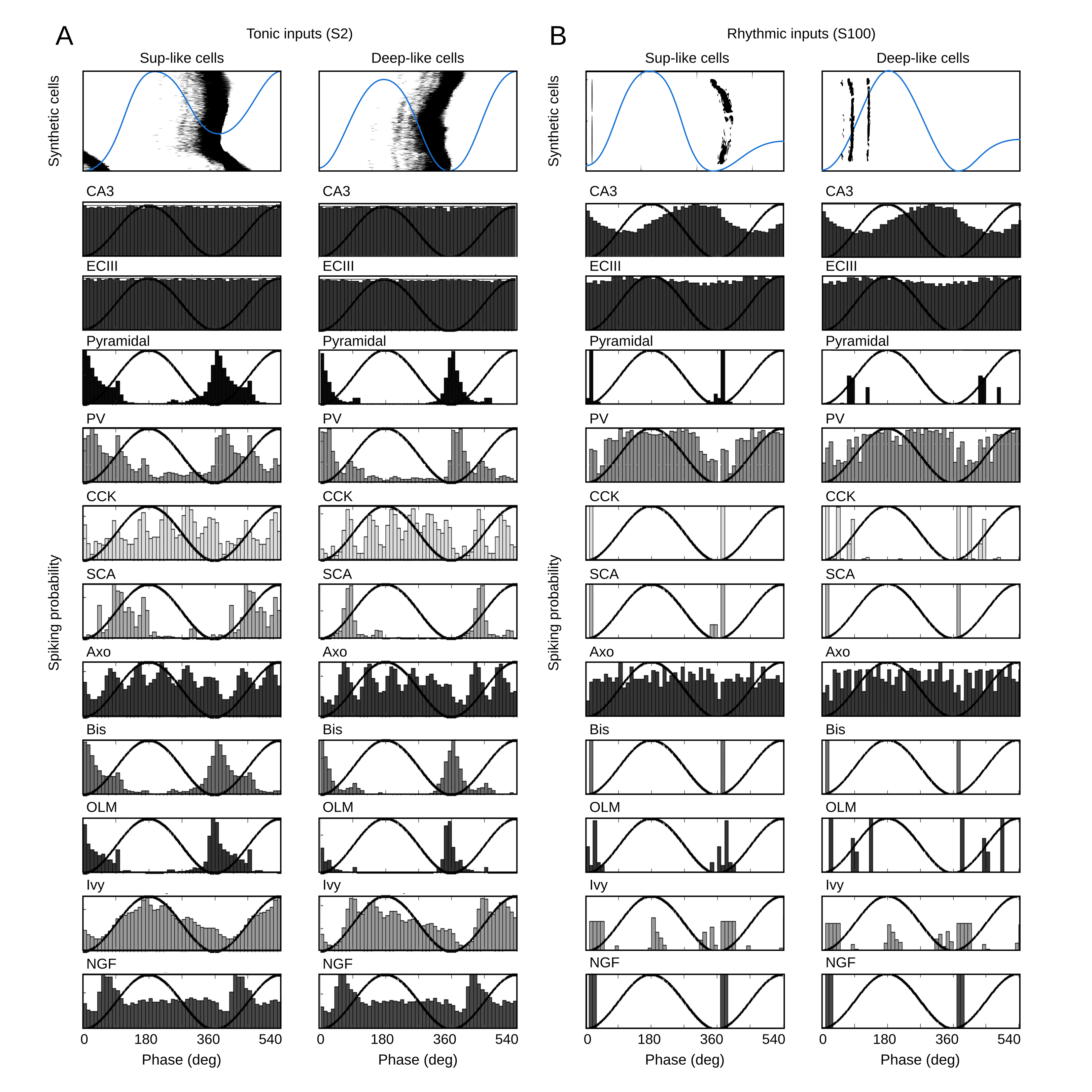


**Supplementary Figure 5. Comparison with the full model.** **A**, Simulation results of superficial- and deep-like parameters in the full model (scale 2) run at the Neuroscience Gateway (NSG) supercomputers (total simulation time: 2000 ms). Note that this model generates theta oscillations autonomously in response to tonic inputs. Using deep- and superficial-like PV/CCK connectivity yielded theta oscillations at similar frequencies: 12 Hz for superficial-like cells and 11 Hz for deep-like cells. Since the LFP signal in the full model is calculated from pyramidal cell activity, the effect of phase-locking preference cannot be tested. Pyramidal cells (n=155750), PV basket cells (n=2765), CCK basket cells(n=1800), SCA interneurons (n=200), Axo-axonic interneurons (n=735), Bistratified cells (n=1105), OLM cells (n=820), Ivy cells (n=4405), NGF cells (n=1790). **B**, Simulation results of superficial- and deep-like parameters in the full model (scale 100) for rhythmic inputs (200 ms; 2 theta cycles). In this simulation glutamatergic CA3 and ECIII inputs were theta-modulated to provide with a general clock and LFP-like theta signal. Using superficial- and deep-like connectivity resulted in different phase-locking preference, similar to our simulation results. Note that phase-locked firing by PV and CCK basket cells differ from the original simulations. Pyramidal cells (n=3115), PV basket cells (n=55), CCK basket cells (n=36), SCA cells (n=4), Axo-axonic cells (n=14), Bistratified cells (n=22), OLM cells (n=16), Ivy cells (n=88), NGF cells (n=35).


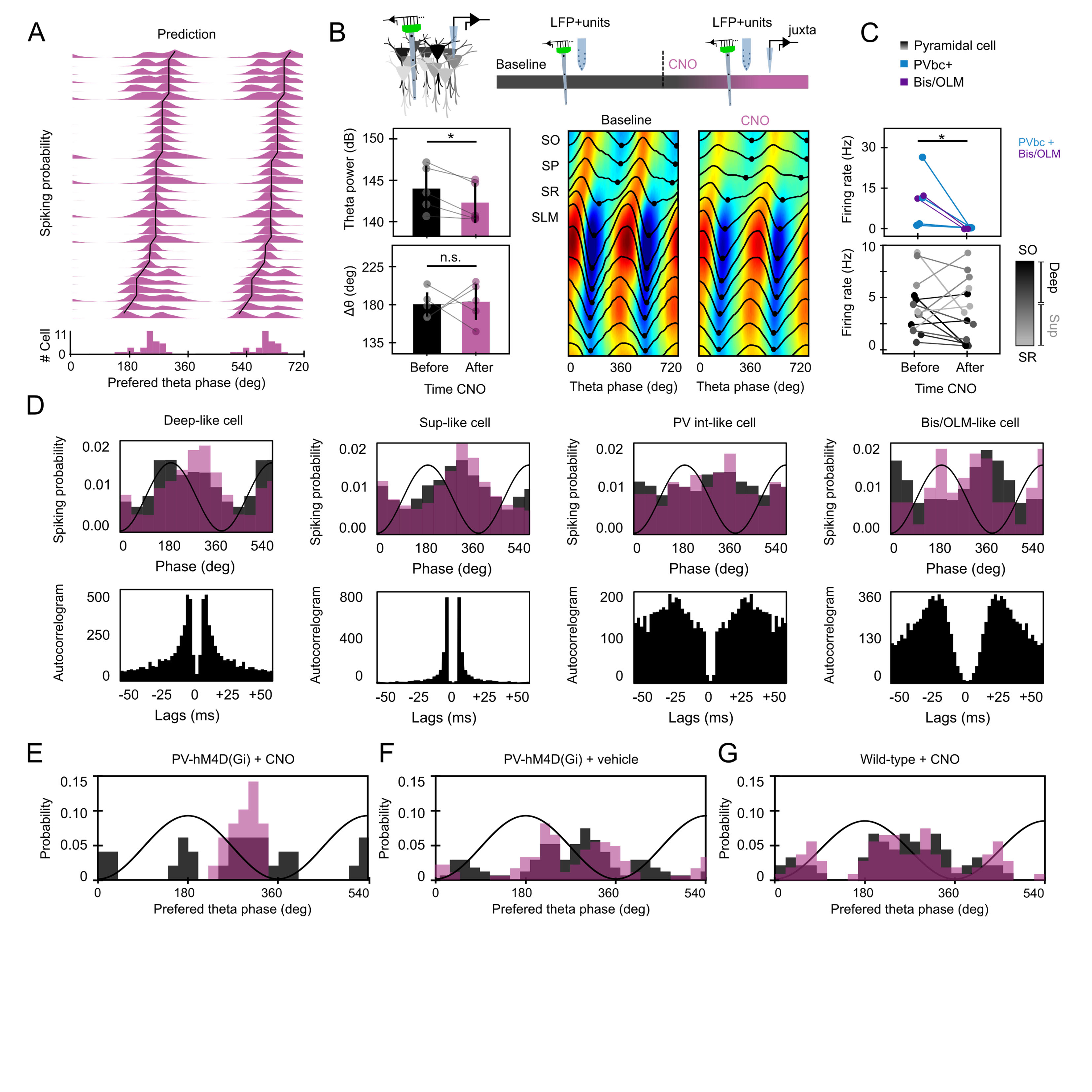


**Supplementary Figure 6. Effects of chemogenetic silencing of PV cells. A,** Model prediction of the effect of blocking PV basket cell inputs in synthetic pyramidal cells (n=32). **B**, Top, Schematic representation of chemogenetic experiments. LFP and multisite recordings were obtained before and after CNO i.p. injections. A subset of individual units recorded with high-density probes could be tracked continuously before and after using kilosort2. Juxtacellular recordings were obtained only after CNO to cope with mechanical stability issues. CNO injection in PV-hM4D(Gi) mice induced a significant reduction of theta power (paired t-test, t(4)=3.9975, p = 0.016; n=5) but not in SLM-SP phase reversal. Bars are mean values +/- SD. Note similar laminar profiles of LFP and CSD theta cycles. CSD profiles are shown in color scale from blue (sink) to red (sources). **C**, Firing rate of the subset of putatively identified cell types evaluated before and after CNO. Putative pyramidal units were classified as putative deep and superficial as in ref.26 (by defining extreme quartiles along the probes as judged by sharp-wave ripple waveforms; n=14). GABAergic units were classified as PV-basket cells, bistratified cells or OLM-like interneurons based on their firing pattern during theta and sharp-wave ripples (see Methods). Note significantly reduction of PV- and Bis/OLM-like cells (paired t-test for all cells together; t(5) = 2.8204, p = 0.0371; n=6), consistent with anatomical confirmation of hM4D(Gi) expression (see Fig.3C). **D**, Theta-phase firing probability before and after CNO (top plots) for individual examples of different cell types recorded in PV-hM4D(Gi) mice. Bottom plots show the autocorrelogram of each unit. **E**, Theta-phase firing probability from all putative pyramidal units isolated before and after CNO (n=12 units) in 2 PV-hM4D(Gi) mice (F(1,22)=4.5, p=0.046). Note bimodal distribution before (significant sublayer effect, F(1,8)=7.2, p=0.028), but not after (no sublayer effect, F(1,8)=0.07, p=0.79). **F**, Theta-phase firing probability from all putative pyramidal units isolated independently before (n=45) and after vehicle (n=47) in 2 PV-hM4D(Gi) mice (F(1,90)=1.8, p=0.18). Similar sublayer effects before, F(1,46)=4.3, p=0.04), and after (F(1,49)=5.2, p=0.02)). **G**, Theta-phase firing histograms from all putative pyramidal units isolated independently before (n=27) and after CNO (n=32) in 3 wild-type mice (F(1,57)=0.3, p=0.6). Similar sublayer effects before (F(1,13)=5.3, p=0.038) and after (F(1,18), p=0.035) CNO. Pre-treatment probability distribution in E, F and G are similar as tested with one-way ANOVA F(2,83)=0.72, p=0.485.


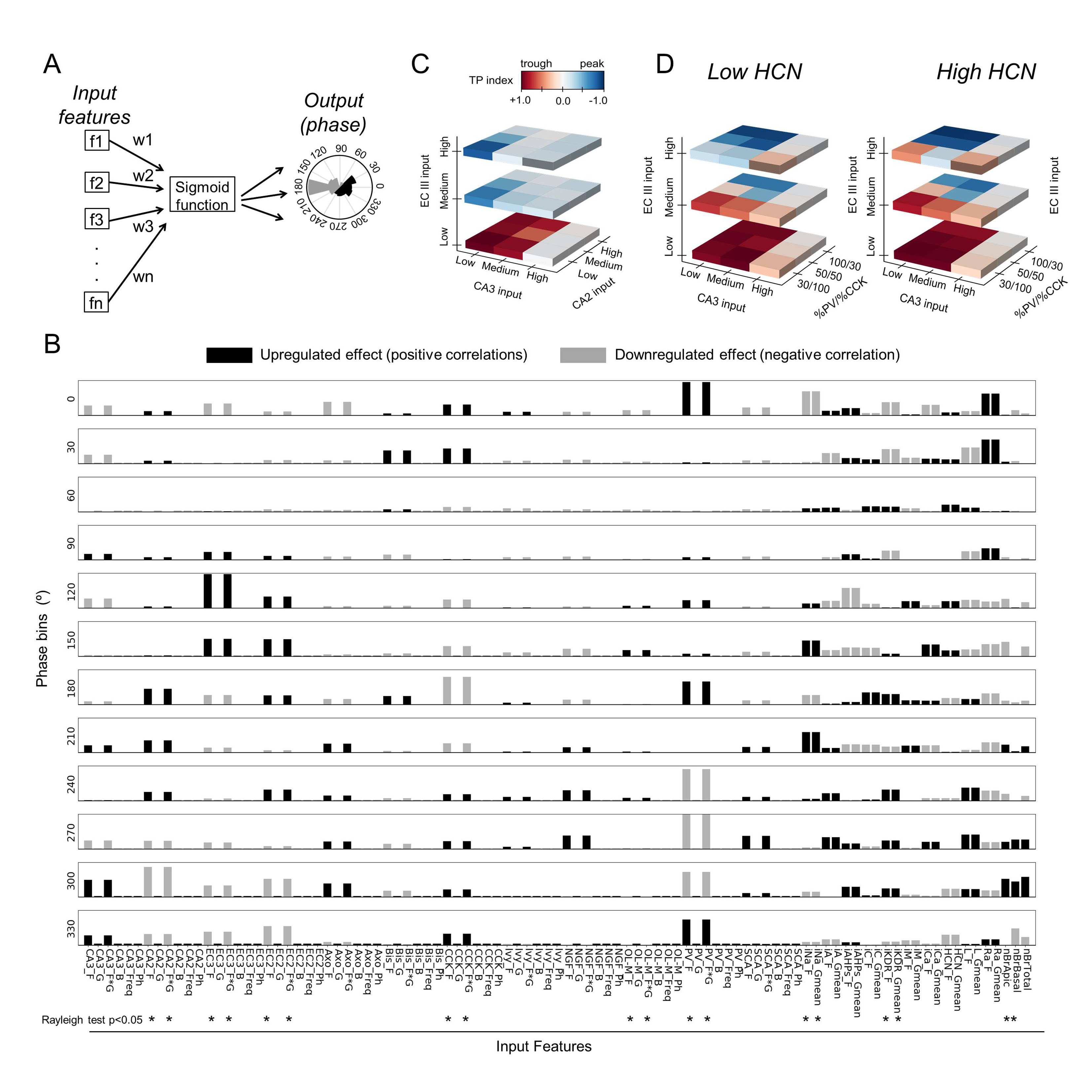


**Supplementary Figure 7. Logistic regression model. A**, A multinomial logistic regression model was implemented to evaluate the relative contribution of different biophysical, intrinsic and connectivity factors (97 input features) in determining phase timing using 731 heterogeneous synthetic cells and more than 300.000 theta cycles. **B**, Results from all contributing factors. Black represents the effect of upregulation; gray represents the effect of downregulation. Asterisks identify factors over the 99% of confidence. **C**, Effect of the interaction between CA3, ECIII and CA2 input pathways in phase shifting phase preference of a representative individual. **D**, Effect of low and high levels of HCN in phase timing individual cells to CA3 and ECIII input pathways as a function of the PV/CCK axis (deep-like: 100% PV-30% CCK; superficial-like: 30% PV-100% CCK).


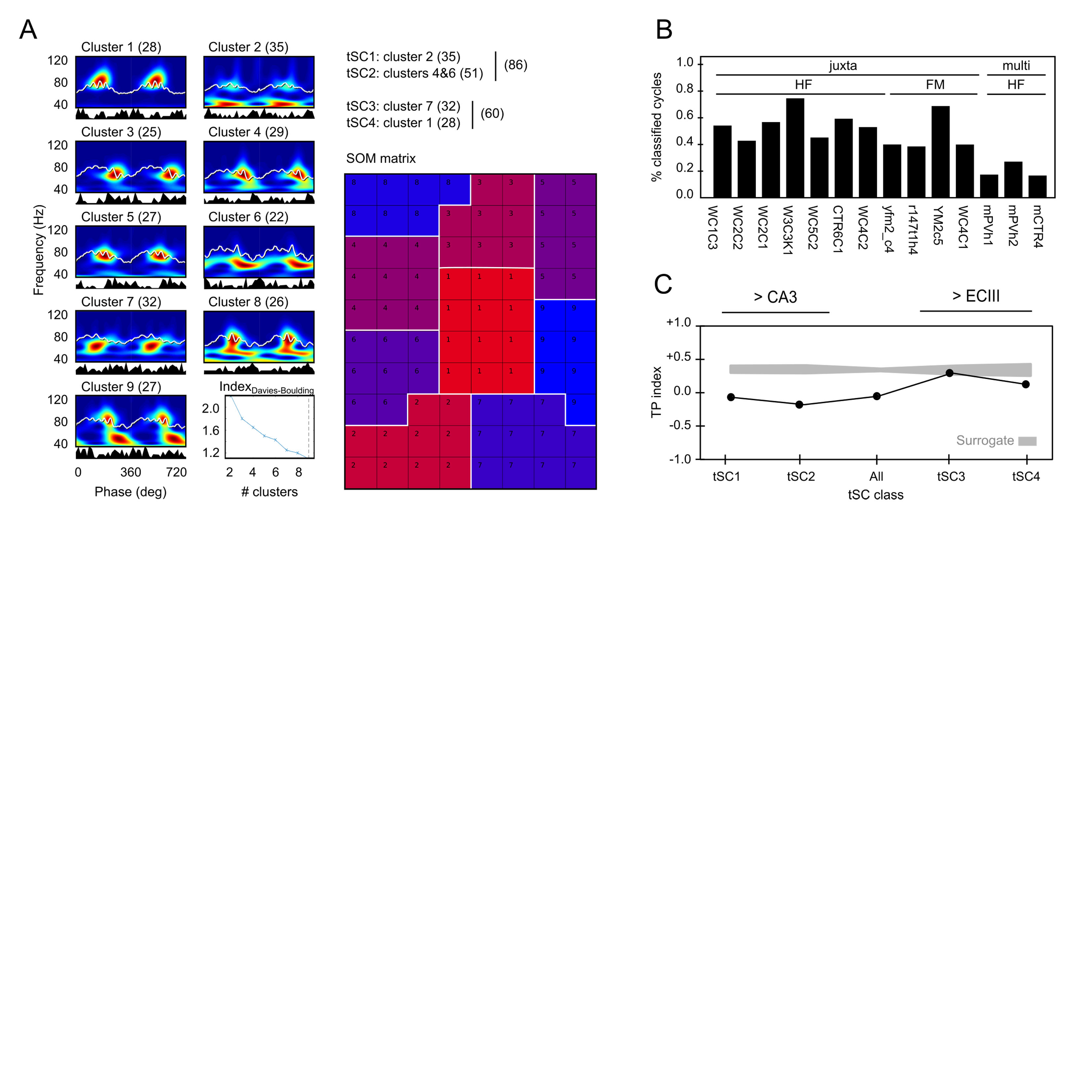


**Supplementary Figure 8. Effect of input pathways on theta phase-locked firing. A**, Clusters of individual theta cycles by SOM are shown together with their mean time-frequency spectra. Number in parentheses refer to the number of individual cycles at each cluster. The SOM matrix at left shows the topological relationship between clusters (indicated by numbers). Theta nested spectral components are blind to SOM. However, since the algorithm clustered cycles based on their filtered waveforms, the different spectral classes (tSC1, 2, 3 and 4) are successfully identified. Note that clusters 4 and 6 were assigned to class tSC2 given their topological proximity and comparable spectral components. The number of clusters was defined by the Davies-Bouldin index. Our Matlab routine for classification of theta cycles, called RhythSOM, is publicly available (<https://github.com/acnavasolive/RhythSOM>). **B**, Percentage of classified theta cycles from the different experiments. HF, head-fixed; FM, freely moving. Total number of cycles from left to right is n=118, 251, 168, 49, 83, 623, 176, 261, 56, 166, 36, 7075, 5264, 35762). **C,** Comparison between TP index values estimated for each tSC class (n(tSC1)=22, n(tSC2)=13, n(tSC3)=19, n(tSC4)=9, n(All)=118) and those obtained from 1000 surrogates (gray) for the cell shown in Fig.5.

| **Parameter** | **Fitting strategy** | **Additional info** |
| --- | --- | --- |
| **Active intrinsic parameters** | | |
| 1. A conductance factor | Fitted by GA | Table S2 |
| 1. AHPs conductance factor | Fitted by GA | Table S2 |
| 1. C conductance factor | Fitted by GA | Table S2 |
| 1. Ca L conductance factor | Fitted by GA | Table S2 |
| 1. Ca T conductance factor | Fitted by GA | Table S2 |
| 1. KDR conductance factor | Fitted by GA | Table S2 |
| 1. M conductance factor | Fitted by GA | Table S2 |
| 1. Na conductance factor | Fitted by GA | Table S2 |
| 1. HCN conductance factor | Fitted by GA | Table S2 |
| **Passive parameters** | | |
| 1. Ra Axial resistivity factor | Fitted by GA | Table S2 |
| 1. Leak conductance factor | Fitted by GA | Table S2 |
| **Synaptic parameters** | | |
| 1. CA3 conductance factor | Fitted by GA | Table S3 |
| 1. CA2 conductance factor | Random value from interval | Table S3 |
| 1. ECIII conductance factor | Random value from interval | Table S3 |
| 1. ECII conductance factor | Random value from interval | Table S3 |
| 1. Axo conductance factor | Fitted by GA | Table S3 |
| 1. Bis conductance factor | Fitted by GA | Table S3 |
| 1. CCK conductance factor | Fitted by GA | Table S3 |
| 1. Ivy conductance factor | Random value from interval | Table S3 |
| 1. NGF conductance factor | Random value from interval | Table S3 |
| 1. OLM conductance factor | Random value from interval | Table S3 |
| 1. PV conductance factor | Fitted by GA | Table S3 |
| 1. SCA conductance factor | Fitted by GA | Table S3 |

**Supplementary Table 1. List of free parameters and fitting strategy used**

**Supplementary Table 2 Active and passive model parameters taken from literature.**

| **Channel/Parameter** | **Location** | **Distance to soma (um)** | | **Vrev (mV)** | **tau (ms)** | **Gmax (S cm^-2)** | **Reference ModelDB** | **GA factor interval** |
| --- | --- | --- | --- | --- | --- | --- | --- | --- |
| ***Active channels*** | | | | | | | | |
| Type A | Soma | all | | -80 | 0,1 | 0.0025 | Ref.69 ModelDB 181967 | (0.1, 1) |
| Type A | Basal | all | | -80 | 0,1 | 0.0600 |  |  |
| Type A | Apic | 50 - 350 | | -80 | 0,1 | 0.0600 |  |  |
| AHPslow | Soma | all | | -80 |  | 0.0005 | Ref. 69 ModelDB 181967 | (0.5, 20) |
| AHPslow | Basal | all | | -80 |  | 0.0005 |  |  |
| AHPslow | Apic | 50 - 350 | | -80 |  | 0.0005 |  |  |
| Type C | Soma | all | | -80 |  | 0.09075 | Ref.69 ModelDB 181967 | (0.5, 20) |
| Type C | Basal | all | | -80 |  | 0.03300 |  |  |
| Type C | Apic | 50 - 200 | | -80 |  | 0.03300 |  |  |
| Type C | Apic | 350 | | -80 |  | 0.00410 |  |  |
| CaL | Soma | all | | 140 | 50 | 0.000700000 | Ref. 70 ModelDB 206244 | (0.5, 20) |
| CaL | Basal | all | | 140 | 50 | 0.000031635 |  |  |
| CaL | Apic | 50 - 350 | | 140 | 50 | 0.000031635 |  |  |
| CaT | Soma | all | | 140 | 50 | 0.00005 | Ref. 70 ModelDB 206244 | (0.5, 20) |
| CaT | Basal | all | | 140 | 50 | 0.00001 |  |  |
| CaT | Apic | 50 - 350 | | 140 | 50 | 0.00001 |  |  |
| KDR | Soma | all | | -80 | 3,5 | 0.001400 | Ref.69 ModelDB 181967 | (0.5, 20) |
| KDR | Axon | all | | -80 | 3,5 | 0.020000 |  |  |
| KDR | Basal | all | | -80 | 3,5 | 0.000868 |  |  |
| KDR | Apic | 50 - 800 | | -80 | 3,5 | 0.000868 |  |  |
| Type M | Soma | all | | -80 |  | 0.06 | Ref. 69 ModelDB 181967 | (0.5, 20) |
| Type M | Axon | all | | -80 |  | 0.03 |  |  |
| Type M | Basal | all | | -80 |  | 0.06 |  |  |
| Type M | Apic | 50 - 350 | | -80 |  | 0.06 |  |  |
| Na | Soma | all | | 40 |  | 0.07 | Ref.69 ModelDB 181967 | (1, 10) |
| Na | Axon | all | | 40 |  | 0.07 |  |  |
| Na | Basal | all | | 40 |  | 0.07 |  |  |
| Na | Apic | 50 - 800 | | 40 |  | 0.07 |  |  |
| HCN | Apic | all | | -30 |  | 0.0000085 | Ref.50 | (0.5, 20) |
| ***Passive properties*** | | | | | | | | |
| Leak (L) | All | all | | -54,3 |  | 0,0025 | Ref.50 | (0.5, 10) |
| Axial resistivity (Ohm) | All | | all |  |  | 100 | Ref.50 | (0, 1) |

**Supplementary Table 3 Synaptic model parameters taken from literature.**

| **Pre-synaptic Cell** | **# boutons** | **Location** | | **Distance to soma (um)** | **Frequency (Hz)** | **E_rev_ (mV)** | **tau_1_ (ms)** | **tau_2_ (ms)** | **Gmax (nS)** | **% Basal firing** | **Phase (deg)** | ***β*_1_** | ***β*_2_** | **References** | **GA interval** |
| --- | --- | --- | --- | --- | --- | --- | --- | --- | --- | --- | --- | --- | --- | --- | --- |
| CA3 | 6209 | Apic | SLM | 50 to 300 | 1.5 | 0 | 0.5 | 3 | 0.0002 | 0.5 | 276 | 5 | 3 | * | (0.5, 20) |
| CA3 | 2661 | Apic | SR (thick) | 50 to 300 | 1 | 0 | 0.5 | 3 | 0.0002 | 0.5 | 276 | 5 | 3 |  |  |
| CA3 | 34 | Apic | SLM | 300 to 600 | 1 | 0 | 0.5 | 3 | 0.0002 | 0.5 | 276 | 5 | 3 |  |  |
| CA3 | 3000 | Axon | SP | -200 to 0 | 1 | 0 | 0.5 | 3 | 0.0002 | 0.5 | 276 | 5 | 3 |  |  |
| CA2 | 8000 | Basal | SO | -200 to 0 | 1 | 0 | 0.5 | 3 | 0.0001-0.004 | 0.25 | 330 | 5 | 5 | * and Ref.56 | N/A. See Gmax ranges |
| ECIII | 968 | Apic | SR (thick) | 300 to 600 | 1 | 0 | 0.5 | 3 | 0.0001- | 0.8 | 180 | 3 | 5 | * | N/A. See Gmax ranges |
| ECIII | 774 | Apic | SLM | 300 to 600 | 1.5 | 0 | 0.5 | 3 | 0.004 | 0.8 | 180 | 3 | 5 |  |  |
| ECII | 700 | Apic | SR (thick) | 300 to 600 | 1 | 0 | 0.5 | 3 | 0.0001- | 0.8 | 330 | 5 | 5 | * | N/A. See Gmax ranges |
| ECII | 1042 | Apic | SLM | 300 to 600 | 1.5 | 0 | 0.5 | 3 | 0.004 | 0.8 | 330 | 5 | 5 |  |  |
| Axo | 34 | Axon | SP | -200 to 0 | 20 | -70 | 0.28 | 8.4 | 0.00115 | 0 | 185 | 10 | 6 | * and Ref.73 | (0.001,2) |
| Bis | 53 | Basal | SO | -200 to -50 | 40 | -70 | 0.11 | 9.70 | 0.00051 | 0 | 1 | 5 | 5 | * and Ref.74 | (0.5, 50) |
| Bis | 3 | Basal | SO | -50 to 0 | 40 | -70 | 0.11 | 9.70 | 0.00051 | 0 | 1 | 5 | 5 |  |  |
| Bis | 4 | Apic | SR (thin) | 0 to 150 | 40 | -70 | 0.11 | 9.70 | 0.00051 | 0 | 1 | 5 | 5 |  |  |
| Bis | 44 | Apic | SR (thin) | 0 to 300 | 40 | -70 | 0.11 | 9.70 | 0.00051 | 0 | 1 | 5 | 5 |  |  |
| CCK | 37 | Basal | SO | -50 to 0 | 60 | -70 | 0.2 | 4.2 | 0.00052 | 0.25 | 174 | 3 | 5 | * and Ref.75 | (0.5, 20) |
| CCK | 37 | Apic | SR (thick) | 0 to 150 | 60 | -70 | 0.2 | 4.2 | 0.00052 | 0.25 | 174 | 3 | 5 |  |  |
| CCK | 30 | Soma | SP | -10 to 10 | 60 | -70 | 0.2 | 4.2 | 0.00052 | 0.25 | 174 | 3 | 5 |  |  |
| CCK | 1 | Apic | SR (thick) | 300 to 600 | 60 | -70 | 0.2 | 4.2 | 0.00052 | 0.25 | 174 | 3 | 5 |  |  |
| Ivy | 169 | Basal | SO | -200 to -50 | 4 | -70 | 1.1 | 11 | 0.00004- | 0.5 | 31 | 3 | 5 | * and ref.76 | N/A. See Gmax ranges |
| Ivy | 4 | Basal | SO | -50 to 0 | 4 | -70 | 1.1 | 11 | 0.00082 | 0.5 | 31 | 3 | 5 |  |  |
| Ivy | 4 | Apic | SR (thin) | 0 to 150 | 4 | -70 | 1.1 | 11 |  | 0.5 | 31 | 3 | 5 |  |  |
| Ivy | 211 | Apic | SR (thin) | 0 to 300 | 4 | -70 | 1.1 | 11 |  | 0.5 | 31 | 3 | 5 |  |  |
| Ivy | 34 | Apic | SLM | 300 to 600 | 4 | -70 | 1.1 | 11 |  | 0.5 | 31 | 3 | 5 |  |  |
| NGF | 24 | Apic | SR (thin) | 0 to 300 | 4 | -70 | 9 | 39 | 0.00003- | 0 | 196 | 4 | 8 | * and Ref.77 | N/A. See Gmax ranges |
| NGF | 116 | Apic | SLM | 300 to 600 | 4 | -70 | 9 | 39 | 0.00013 | 0 | 196 | 4 | 8 |  |  |
| OLM | 5 | Basal | SO | -200 to -50 | 23 | -70 | 0.13 | 11 | 0.00015- | 0.1 | 19 | 6 | 6 | * and Ref.78 | N/A. See Gmax ranges |
| OLM | 72 | Apic | SLM | 300 to 600 | 23 | -70 | 0.13 | 11 | 0.006 | 0.1 | 19 | 6 | 6 |  |  |
| PV | 61 | Basal | SO | -50 to 0 | 50 | -70 | 0.3 | 6.2 | 0.0002 | 0.3 | 271 | 15 | 10 | * | (0.5, 20) |
| PV | 61 | Apic | SR (thick) | 0 to 150 | 50 | -70 | 0.3 | 6.2 | 0.0002 | 0.3 | 271 | 15 | 10 |  |  |
| PV | 61 | Soma | SP | -10 to 10 | 50 | -70 | 0.3 | 6.2 | 0.0002 | 0.3 | 271 | 15 | 10 |  |  |
| SCA | 1 | Basal | SO | -200 to -50 | 7 | -70 | 0.3 | 8 | 0.00037 | 0 | 205 | 5 | 5 | * | (0.5, 20) |
| SCA | 12 | Apic | SR (thin) | 0 to 300 | 7 | -70 | 0.3 | 8 | 0.00037 | 0 | 205 | 5 | 5 |  |  |
| SCA | 1 | Apic | SR (thick) | 300 to 600 | 7 | -70 | 0.3 | 8 | 0.00037 | 0 | 205 | 5 | 5 |  |  |

* Ref. 11, 33, 46 and 76

**Supplementary Table 4. Model validation of measurements not-fitted by GA**

| **Measurements** | **Experimental values/ References** | | **Model** |
| --- | --- | --- | --- |
| ***Intrinsic properties (evaluated at -0.1-0.5 nA)*** | | | |
| After-hyperpolarization (mV) | -3.3 ± 1.7 (superficial)  -3.8 ± 2.2 (deep)  From -1.29 to -1.39 | Our lab (in vivo). Ref.36  n=10 sup, n=11 deep  Ref.80 (in vitro) | -1.6 ± 2.6 |
| After-depolarization  (mV) | 0.38 ± 0.26 (superficial)  0.43 ± 0.35 (deep)  From 5.2 to 8.4 | Our lab (in vivo). Ref.36  n=10 sup, n=11 deep  Ref.80 (in vitro) | 0.3 ± 0.7 |
| Sags  (mV) | 1.0 ± 0.8 (superficial)  0.4 ± 0.1 (deep)  4.2 ± 0.2 (superficial)   - 1. ± 0.1 (deep) | Our lab (in vivo). Ref.36  n=10 sup, n=11 deep  Ref.51 (in vitro) | -0.3 ± 0.7 |
| Action potential duration (ms) | 1.02 ± 0.13 (superficial)  0.96 ± 0.14 (deep) | Our lab (in vivo). Ref.36  n=10 sup, n=11 deep | 0.9 ± 0.3 |
| Action potential threshold (mV) | -51.8 ± 4.4 (superficial)  -50.0 ± 6.7 (deep) | Our lab, unpublished  n=10 sup, n=11 deep | -55.0 ± 9.2 |
| ***Oscillatory properties*** | | | |
| Preferred theta phase (deg) | 354.9 ± 24.3 (superficial)  175.1 ± 65.3 (deep)  116.1 ± 68.3 (superficial)  160.6 ± 68.4 (deep) | This study (head-fixed)  n=5 sup, n=6 deep  This study (freely-moving)  n=7 sup, n=13 deep | 293.5 ± 26.3 (sup-like)  198.7 ± 19.2 (deep-like) |
| Mean vector length | 0.20 +/- 0.06 (superficial)  0.24 +/- 0.09 (deep)  0.21 +/- 0.06 (superficial)  0.39 +/- 0.14 (deep) | This study (Head-fixed)  n=5 sup, n=6 deep  This study (Freely-moving)  n=7 sup, n=13 deep | 0.8 ± 0.1 (sup-like)  0.8 ± 0.1 (deep-like) |

In vivo data from our lab was partially reported in Ref.36. Data was reanalyzed to provide experimental ranges for deep and superficial cells. See also <http://hippocampome.org/php/index.php>

***** Values of after-hyperpolarization (AHP) and depolarization (ADP) were estimated from Ref.80 as AHP/ADP – (Spike threshold – Resting Membrane potential).
