# Supplementary material for: Multimodal determinants of phase-locked dynamics across deep-superficial hippocampal sublayers during theta oscillations
Source: Nat Commun. 2020 May 5;11:2217. doi: 10.1038/s41467-020-15840-6 (PMC7200700; doi:10.1038/s41467-020-15840-6)
Supplement: Supplementary file 3 — Reporting Summary [file 41467_2020_15840_MOESM3_ESM.pdf]

nature research

Corresponding author(s): Liset M de la Prida

Last updated by author(s): Mar 23, 2020

Reporting Summary

Nature Research wishes to improve the reproducibility of the work that we publish. This form provides structure for consistency and transparency in reporting. For further information on Nature Research policies, see Authors & Referees and the Editorial Policy Checklist .

Statistics

For all statistical analyses, confirm that the following items are present in the figure legend, table legend, main text, or Methods section.

n/a	Confirmed	
☐	☒	The exact sample size (*n*) for each experimental group/condition, given as a discrete number and unit of measurement
☐	☒	A statement on whether measurements were taken from distinct samples or whether the same sample was measured repeatedly
☐	☒	The statistical test(s) used AND whether they are one- or two-sided<br>*Only common tests should be described solely by name; describe more complex techniques in the Methods section.*
☐	☒	A description of all covariates tested
☐	☒	A description of any assumptions or corrections, such as tests of normality and adjustment for multiple comparisons
☐	☒	A full description of the statistical parameters including central tendency (e.g. means) or other basic estimates (e.g. regression coefficient) AND variation (e.g. standard deviation) or associated estimates of uncertainty (e.g. confidence intervals)
☐	☒	For null hypothesis testing, the test statistic (e.g. *F*, *t*, *r*) with confidence intervals, effect sizes, degrees of freedom and *P* value noted<br>*Give P values as exact values whenever suitable.*
☒	☐	For Bayesian analysis, information on the choice of priors and Markov chain Monte Carlo settings
☐	☒	For hierarchical and complex designs, identification of the appropriate level for tests and full reporting of outcomes
☐	☒	Estimates of effect sizes (e.g. Cohen's *d*, Pearson's *r*), indicating how they were calculated

Our web collection on statistics for biologists contains articles on many of the points above.

Software and code

Policy information about availability of computer code

Data collection	Open ephys, Axoscope (v 11), Multichannel Systems MC Rack (v 4.6.2)
Data analysis	Matlab 2016b, Neuron (v.7.4) + Python (v.7) , ImageJ (v 1.52a), Leica LAS AF software v2.6.0, CircStat Matlab toolbox, CA1 pyramidal cell model is available at Github https://github.com/acnavasolive/LCN-HippoModel, Morphologies were obtained from http://neuromorpho.org/ (morphologies n128, n127 and n409 from the Turner archive and morphology sup1 corresponding to the superficial cell I040913C7SEC8_2D from the Prida archive), Our theta cycle classifier RhythSOM is available at GitHub https://github.com/acnavasolive/RhythSOM; Kilosort2 (https://github.com/MouseLand/Kilosort2)

For manuscripts utilizing custom algorithms or software that are central to the research but not yet described in published literature, software must be made available to editors/reviewers. We strongly encourage code deposition in a community repository (e.g. GitHub). See the Nature Research guidelines for submitting code & software for further information.

Data

Policy information about availability of data

All manuscripts must include a data availability statement. This statement should provide the following information, where applicable:

- Accession codes, unique identifiers, or web links for publicly available datasets
- A list of figures that have associated raw data
- A description of any restrictions on data availability

Experimental data supporting the findings of this study are available on request from the corresponding author.

Field-specific reporting

Please select the one below that is the best fit for your research. If you are not sure, read the appropriate sections before making your selection.

- [x] Life sciences
- [ ] Behavioural & social sciences
- [ ] Ecological, evolutionary & environmental sciences

For a reference copy of the document with all sections, see nature.com/documents/nr-reporting-summary-flat.pdf

Life sciences study design

All studies must disclose on these points even when the disclosure is negative.

Sample size	No statistical method was used to predetermine sample sizes. Juxtacellular recordings fulldataset: n=12 cells from 12 head-fixed mice; n=28 cells from 28 freely moving rats. Opto-tagging experiments: n=7 units from 3 Calb1+AAV-ChR2 mice, n=14 units from 2 Thy1-ChR2 mice. Chemogenetic experiments: n=5 juxtacellularly recorded cells from 5 PV-hM4D(Gi) mice, n=23 units from 2 PV-hM4D(Gi) mice treated with CNO; n=45 units in 2 PV-hM4D(Gi) mice treated with vehicle; n=32 units from 3 wild-type mice treated with CNO; Multi-site and theta cycle experiments: n=96 units from 3 mice (2 PV-Cre mice, 1 wild-type mouse)
Data exclusions	For theta phase preference analysis cells should be significantly modulated (Rayleigh test p<0.05). For morphological analysis, only cells sucessfully recovered from labeling were included.
Replication	The exact number of replications for each experiment is detailed in text and figures. Juxtacellular recordings were replicated in 12 head-fixed mice and n=28 freely moving rats. Opto-tagging experiments were replicated in 3 CaPV-hM4D(Gi) mice withlb1+AAV-ChR2 and 2 Thy1-ChR2 mice.. Chemogenetic experiments were replicated in 7 PV-hM4D(Gi) mice with CNO and 2 PV-hM4D(Gi) mice with vehicle. Effects of CNO were replicated in 3 wild-type mice. Multi-site and theta cycle experiments were replicated in 3 mice (2 PV-Cre mice, 1 wild-type mouse). All attempts of replication were succesfull.
Randomization	Animals were allocated randomly to the different experimental groups. Randomization is included for data analysis and described accordingly. For computer simulation we reamdomly selected different sets of GA factors. Synthetic cells used for analysis in Fig.2, 3 and 4 were randomly selected from the pool of validated cells. Surrogate tests for theta cycle subclasess were run for 1000 shuffles. Significant rank order distributions were tested against 500 shuffles.
Blinding	Ressearchers were blinded during cell recordings. Histological analysis was blind to electrophysiology. Computational model was blind to electrophysiological data.

Reporting for specific materials, systems and methods

We require information from authors about some types of materials, experimental systems and methods used in many studies. Here, indicate whether each material, system or method listed is relevant to your study. If you are not sure if a list item applies to your research, read the appropriate section before selecting a response.

Materials & experimental systems

n/a	Involved in the study	
[ ]	[x]	Antibodies
[x]	[ ]	Eukaryotic cell lines
[x]	[ ]	Palaeontology
[ ]	[x]	Animals and other organisms
[x]	[ ]	Human research participants
[x]	[ ]	Clinical data

Methods

n/a	Involved in the study	
[x]	[ ]	ChIP-seq
[x]	[ ]	Flow cytometry
[x]	[ ]	MRI-based neuroimaging

Antibodies

Antibodies used	Rabbit anti-calbindin D-28k Swant Swant CB-38; RRID:AB_10000340;<br>Mouse anti-calbindin D-28k Swant Swant 300; RRID:AB_10000347<br>Goat anti-rabbit Alexa Fluor633 IgG Invitrogen Cat# A21070; RRID:AB_2535731<br>Goat anti-mouse Rhodamine Red IgG Jackson Immunoresearch Cat# 115-295-003; RRID:AB_2338756<br>Alexa Fluor488-conjugated streptavidin Jackson Immunoresearch Cat# 016-540-084, RRID:AB_2337249<br>Rabbit anti-somatostatin (1:1000, Peninsula T4103, RRID:AB_518614),<br>Rabbit anti-PV (1:1000, Swant AB_2631173, RRID:AB_2631173),<br>Mouse anti-PV (1:1000, Swant AB_10000343, RRID:AB_10000343),<br>Rabbit anti-NPY (1:1000; Peninsula, T4070, RRID: AB_518504)
Validation	Method specificity was tested by omitting primary antibodies and using secondary antibodies with similar imaging parameters as

in the full reaction. Specific information for primary antibodies: CB, rabbit polyclonal produced by immunization with recombinant rat CB D-28k, antiserum; characterized in rat; no signal in mouse knockout. CB, mouse monoclonal from chicken CB D-28k similar immunoreactivity as for the rabbit antibody. Rabbit anti-PV antibody as in PMID 32016558. Mouse anti-PV antibody showed similar immunoreactivity. Rabit anti-somatoostatin antibody similar labeling as a rat antibody Chemicon MAB354 PMID 11074441. Rabbit anti-NPY labeling similar to that reported in PMID 22666191

Animals and other organisms

Policy information about studies involving animals; ARRIVE guidelines recommended for reporting animal research

Laboratory animals	Mus Musculus and Wistar Rat. A total of 25 males and females mice were used from wild-type (C57BL/6J, n=15), PV-Cre (B6;129P2-Pvalbtm1(cre)Arbr/J; Jackson labs, n=9), Calb1-Cre (Calb1-2A-dgCre-D; Jackson labs, n=3) and Thy1-ChR2-YFP (B6.Cg-Tg(Thy1-COP4/EYFP)18Gfng/J, Jackson labs; n=2) lines. We also used 28 wild-type males and females Wistar rats. Animals were maintained in a 12 h light-dark cycle (7am to 7pm) with access to food and drink ad libitum.
Wild animals	This study does not involve wild animals
Field-collected samples	No field collected samples were used in the study
Ethics oversight	All protocols and procedures were performed according to the Spanish legislation (R.D. 1201/2005 and L.32/2007) and the European Communities Council Directive 2003 (2003/65/CE) for animal research. Experiments were approved by the Ethics Committee of the Instituto Cajal (CSIC). A number of recordings in freely moving rats were obtained at the University of Szeged, Hungary and were approved by the Animal Care Committee of the University of Szeged.

Note that full information on the approval of the study protocol must also be provided in the manuscript.
